# Co-Design of a Consultation Audio-Recording Mobile App for People With Cancer: The SecondEars App

**DOI:** 10.2196/11111

**Published:** 2019-03-12

**Authors:** Ruby Lipson-Smith, Fiona White, Alan White, Lesley Serong, Guy Cooper, Georgia Price-Bell, Amelia Hyatt

**Affiliations:** 1 Cancer Experiences Research Peter MacCallum Cancer Centre Melbourne, Victoria Australia; 2 Wave Digital Melbourne, Victoria Australia

**Keywords:** referral and consultation, adult, humans, cancer, audiovisual aids, mobile apps, community-based participatory research, health behavior, psychological theory

## Abstract

**Background:**

Many patients choose to audio-record their medical consultations so that they can relisten to them at home and share them with family. Consultation audio-recordings can improve patients’ recall and understanding of medical information and increase their involvement in decision making. A hospital-endorsed consultation audio-recording mobile app would provide patients with the permission and means to audio-record their consultations. The Theory of Planned Behavior provides a framework for understanding how patients can be encouraged to appropriately audio-record consultations.

**Objective:**

The aim of this study was to use a co-design process to develop a consultation audio-recording mobile app called *SecondEars*.

**Methods:**

App development began with stakeholder engagement, followed by a series of 6 co-design workshops and then user acceptance testing. Stakeholder engagement included advice from legal, information technology (IT), clinical and allied health leads; digital strategy; and medical records. he co-design workshops were attended by: patient consumers, members of the research team, IT staff, the app designers, clinicians, and staff from medical records. During workshops 1 to 4, the purpose and scope of the app were refined, possible pitfalls were addressed, and design features were discussed. The app designers then incorporated the results from these workshops to produce a wireframe mock-up of the proposed SecondEars app, which was presented for feedback at workshops 5 and 6.

**Results:**

The stakeholders identified 6 requirements for the app, including that it be patient driven, secure, clear in terms of legal responsibilities, linked to the patient’s medical record, and that it should require minimal upfront and ongoing resources. These requirements informed the scope of the co-design workshops. The workshops were attended by between 4 and 13 people. The workshop attendees developed a list of required features and suggestions for user interface design. The app developers used these requirements and recommendations to develop a prototype of the SecondEars app in iOS, which was then refined through user acceptance testing.

**Conclusions:**

The SecondEars app allows patients to have control and autonomy over audio-recording and sharing their consultations while maintaining privacy and safety for medical information and legal protection for clinicians. The app has been designed to have low upkeep and minimal impact on clinical processes. The SecondEars prototype is currently being tested with patients in a clinical setting.

## Introduction

### Background

#### Facilitating Patient-Centered Care

Shared decision making and patient participation are essential elements of patient-centered care [1]. However, patient participation is reduced when patients do not understand or remember information given to them by their health care team [2]. Patients’ ability to retain health care information can be compromised if the patients have low health literacy or language barriers or if the information is complex or distressing [3-5]. Consultation audio-recordings are an effective method to improve patients’ recall and understanding of medical information and subsequently increase their involvement in decision making [6-10].

#### Smartphones for Consultation Audio-Recording

With the increase in smartphone ownership, patients are taking the initiative to audio-record their medical consultations themselves [11]. Recording of consultations is predominantly undertaken because of a desire to increase understanding and to facilitate discussions with family [12], but only a few health care systems have recording policies in place and patients sometimes audio-record consultations without their clinician’s knowledge [13,14]. Patients have therefore self-identified a problem in their care—namely, their lack of understanding and recall of medical information—and a solution to this problem: mobile health (mHealth) technology. Furthermore, patients have expressed a desire for health care providers to institute clear, permissive strategies to facilitate consultation audio-recording [12]. Previous studies have also emphasized the patients’ desire to control which consultations are audio-recorded [5]. Clinicians and health care providers must now work with patients to implement official systems of consultation audio-recording to facilitate an environment where audio-recording is openly encouraged. Medico-legal and trust concerns may be a barrier for some clinical staff regarding participation in consultation audio-recording [15]. An official consultation audio-recording system, such as a suitable smartphone app, may mitigate any potential fear or mistrust that could emerge between the patient and clinician as a result of audio-recordings. A suitable consultation audio-recording smartphone app would promote responsible use of consultation audio-recording, meet relevant legal requirements, and align with patient-centered care by placing control in the hands of the patient.

Previous consultation audio-recording studies have utilized digital recorders or Dictaphones operated by hospital staff, and a copy of the audio-recording was then given to the patient to take home on an audiotape, CD, or USB [16-19]. Dictaphones require resource-intensive setup and maintenance by staff. The administrative load of this system prohibits implementation into usual care, and it prevents patient control over the audio-recording. Clinicians and hospital administrators understand that consultation audio-recordings are beneficial for patients, but they emphasize that successful implementation would require a system that (1) has low upkeep with minimal burden on clinical processes and resources, (2) addresses medico-legal concerns, (3) clearly defines who is responsible for the audio-recording once it is made, and (4) responds to patient preference by allowing the patients to control when they audio-record and with whom they share the audio-recording [11,15].

#### Co-Design of a Smartphone App

If a consultation audio-recording app is to meet the needs of patients, family, clinicians, and hospital administrators, all stakeholders must be involved in the app design and development [20]. Experience-based co-design is the process whereby future end users and other stakeholders draw on their experience and work with designers to design a product or service [21,22]. The experience-based co-design approach applies the key tenets of patient participation. Patients, as experts in their care, are involved in all facets of the project from solution generation, project design and oversight, through to design and testing. Patients have already identified smartphones as a means to audio-record their consultations [11,13,14]. The aim of this study was to use experience-based co-design to design a consultation audio-recording mobile app called *SecondEars* that utilizes this patient-identified solution while working to meet the implementation requirements identified by clinicians and hospital administrators.

### Objectives

The objectives of this co-design study were to:

1. Identify and engage the stakeholders integral to implementation of mHealth technology within a hospital.
2. Facilitate co-design workshops to identify the necessary features of the app.
3. Develop a wireframe of the app.
4. Conduct user acceptance testing of the app.
5. Complete a prototype of the app.

Future publications will report on the piloting and implementation of the SecondEars app into usual care.

## Methods

### Study Design

**Figure 1:**
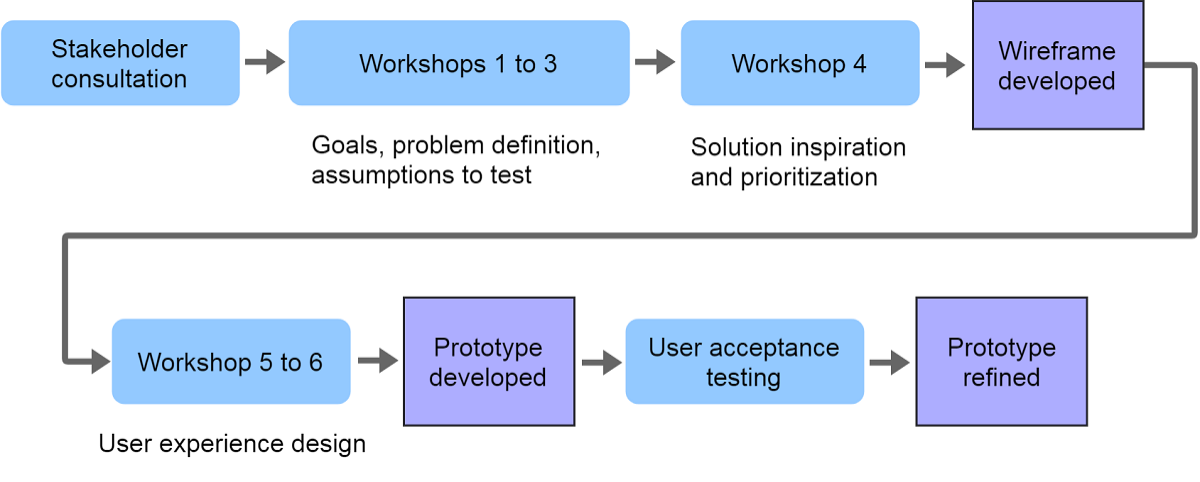
The co-design process for the SecondEars consultation audio-recording app.

This study was conducted at the Peter MacCallum Cancer Centre (Peter Mac) in Melbourne, Australia, and was approved by the relevant ethics committee (reference number: 16/07L). Objectives 1 to 5 were met via the co-design process outlined in Figure 1. All workshop attendees provided written informed consent.

### Theory of Planned Behavior

The Theory of Planned Behavior provided a framework for the design and future implementation of the SecondEars app. The Theory of Planned Behavior posits that a person’s behavior is directly related to the person’s intentions, which in turn result from subjective norms, beliefs, and perceived control over the behavior [23]. If the patients are to be encouraged to responsibly audio-record consultations, the following should be addressed in the design of the SecondEars app:

1. Clinicians’, hospital administrators’, and patients’ beliefs about consultation audio-recording
2. Patients’ perceived control over audio-recording
3. The subjective norms of consultation audio-recording

Apps are user-controlled; it would be up to the patients to download and use an app on their own device, thereby supporting perceived control. An app can be promoted by the hospital, giving patients permission to audio-record openly, thereby developing a subjective norm. An app could also link audio-recordings to the hospital’s medical record or information technology (IT) systems and allow patients to share the audio-recording with family or friends, indicating the safety and utility of audio-recordings, which may change clinicians’, hospital administrators’, and patients’ beliefs about consultation audio-recording.

A well-designed consultation audio-recording mobile app could therefore positively influence behavioral, normative, and control beliefs and, according to the Theory of Planned Behavior, change intentions and encourage responsible audio-recording behavior.

### Stakeholder Engagement

Initial consults with key stakeholders began in 2016 and continued throughout the development process. This early engagement informed the legal and technical requirements of the app before commencing the co-design process (see the Results section for a summary of these requirements). The following 16 stakeholders were consulted: 2 members of the hospital’s legal department, 4 members of the IT department, including the head of department, 5 clinical and allied health leads, the director of digital strategy, the head of the medical records department, and 3 consumer advocates. These stakeholders were kept informed throughout the development process and some attended workshops. The consumer advocates also took a formal place within the project steering committee as associate investigators. The proposed requirements of the app were reviewed and approved by the New Technology Review Committee at Peter MacCallum Cancer Centre in December 2016.

### Developer Engagement

A local mobile app development company, Wave Digital, was contracted in February 2017 to create the SecondEars app. Wave Digital readily embraced the co-design approach and used elements of the Design Sprint methodology to structure the design process [24].

### Co-Design Workshops and Activities

A total of 6 co-design workshops were held between April and June 2017 (see Figure 1). Some of the methods used to elicit information during the workshops included frequently asking open and obvious questions such as “Why.” In addition, rephrasing assumptions or problems as questions was critical to accurately capture the goals for the product and identify the problem the product was attempting to solve.

#### Stage 1: Goals, Problem Definition, and Assumptions to Test (Workshops 1-3)

Before the first workshop, the attendees were briefed on the rationale for developing the SecondEars app, the proposed primary functions of the app, and the requirements and constraints that had been identified by the key stakeholders. The following 3 questions were addressed in workshops 1 to 3:

1. What do we want the app to do?
2. How can we imagine the app failing?
3. Who will be involved in using the app?

To address question 1, the attendees brainstormed a list of functions that the app should be able to do; this list was iteratively added to and refined throughout workshops 1 to 3. To address question 2, the attendees identified possible reasons that the app may fail and then reframed these potential pitfalls as knowledge-seeking questions. These knowledge-seeking questions were used as starting points to generate strategies that could be employed to prevent the potential pitfalls. To define who would be involved in using the app (question 3), a technique called Journey Mapping was used, and the attendees mapped out the pattern of use for the app in the broader context of the patient’s journey through Peter Mac. This included how and when a patient may become aware of it; how and when they may download it; who may encounter the app before, during, or after the patient’s hospital consultation; and who may listen to the audio-recording.

#### Stage 2: Solution Inspiration and Prioritization (Workshop 4)

During workshop 4, attendees compiled a list of existing apps that they believed were well designed, intuitive, or provided a unique experience. These apps were then used to provide inspiration for the user interface design of the SecondEars app.

The co-design team used the MoSCoW method [25] to prioritize the desirable functions that had been identified during workshops 1 to 3. MoSCoW stands for the following: must-haves (Mo), should-haves (S), could-haves (Co), and won't-haves (W). This method allowed the attendees to reach a common understanding of the scope of the project and the relative importance of each of the functions that were listed during workshops 1 to 3.

Following workshop 4, the app developers used a technique called wireframing to do the following:

1. Structure the composition of the features and functions of the app (as prioritized during workshop 4).
2. Prioritize the content on those interfaces.
3. Connect the interfaces into a logical user flow.

#### Stage 3: User Experience Design (Workshops 5 and 6)

In workshop 5, the proposed designs for the app were presented in paper form using a series of interfaces cut to size. The feedback from this workshop informed the wireframes of the app. These wireframes were presented on an iPhone in workshop 6. The app developers conducted one-on-one user experience feedback sessions with each of the attendees to gauge each attendees’ thought process and responses to using this wireframe design. Each attendee was given the wireframe app on an iPhone and asked to complete the following 4 tasks without prompting:

1. Make an audio-recording and then listen back to it.
2. Write a note on one of the recordings.
3. Read previously made notes while recording a consultation.
4. Log out of the app.

### User Interface Design

The final stage of the design process was to create a visual identity for the SecondEars app. The combination of a logo, color, typography, and iconography was developed during the design process. Those brand elements were then applied to the interfaces of the SecondEars app, incorporating all feedback gathered during the sixth co-design workshop.

### Development and User Acceptance Testing

Wave Digital used the finalized visual designs to develop a prototype of the app. Immediate feedback from the research team was incorporated and, in September 2017, the refined prototype was released to the co-design team for user acceptance testing so that any bugs could be identified. All faults were then rectified before the SecondEars app was made available in Test Flight mode on the Apple App Store.

## Results

### Iterative Refinement

Each stage of the development process resulted in requirements and refinements that were incorporated into the final design of the SecondEars app. The outcomes of each stage of the development process are outlined below.

### Requirements Identified Through Stakeholder Engagement

The requirements identified by the stakeholders are outlined in Table 1. These requirements established the design profile and constraints of the SecondEars app and provided a foundation for the workshops.

To meet requirement 6 (see Table 1), the research team chose to develop a minimum viable product and assess its success before making additional financial investment. For this reason, some aspects of requirement 5 were not addressed in this version of the app. Specifically, the research team decided not to automate the app’s connection to the medical record until the app had been piloted within clinical care. If piloting indicates high uptake of the app, then the investment in the IT infrastructure necessary to securely and automatically upload the audio-recordings to the medical record would be justified. In the interim, the audio-recordings would be securely hosted through a cloud solution (Amazon Web Servers) with an interface to allow the medical record staff to access the audio-recordings and manually upload them to the patient’s medical record if necessary, thus meeting requirement 3. The research team identified a potential tension between requirement 1 and requirement 3: if the app is entirely patient driven, then the responsibility of uploading the audio-recording to the medical record falls to the patient. This process could not be automated; therefore, the research team introduced a requirement that all audio-recordings must be uploaded before they can be played back or shared by the patient.

In response to requirement 6, the research team decided to further reduce upfront costs by initially creating the app in iOS for iPhone, not Android. App development in Android is more complex and costly as the app design must be tested on a larger number of Android devices, whereas iOS can be developed for 1 platform only. Developing for iOS first enables more feedback to be gathered from users and any issues addressed before investing in an Android version. It was therefore pragmatic to delay releasing the app in Android until it had been piloted in iOS.

### Workshop Attendees

Each workshop was attended by between 4 and 13 people who together comprised the co-design team. The co-design team included the following: patient consumers, members of the research team, representatives from IT, app designers, clinicians, hospital volunteers, and a representative from the medical records department. Table 2 shows the number and type of attendees at each workshop. One consumer attended 4 of the workshops (female, 56 years of age, previous experience as both a patient and a carer, and self-identified as having intermediate technology skills). Another consumer attended 2 of the workshops (male, 66 years of age, previous experience as both a patient and a carer, and self-identified as having intermediate technology skills). The third consumer attended the final workshop (female, 64 years of age, previous experience as a carer, and self-identified as having beginner technology skills).

**Table 1:** The requirements of the app identified through stakeholder engagement.

Requirement	Description of requirement	Suggested means to meet the requirement
1. Patient-driven	The app should be used by patients, not hospital staff; If the patient’s clinician has given permission to be audio-recorded, the patient should have ultimate control over when and how the patient uses the app; This is not only important in terms of patient participation but also for practicality and financial feasibility of the app (see requirement 5)	The patient must be able to source, download, and use the app independently, with minimal input from hospital staff
2. Secure	The audio-recordings saved on the app and shared from the app must be secure as they will contain identifiable information	Access to recordings should be given only to users of the system via Secure Sockets Layer; The actual recording files should never be sent via unsecure means (eg, short message service, email); Strong password policy for Admin access
3. Linked to medical record	Consultation audio-recordings should be considered a part of the patient’s medical record; Saving original copies of the audio-recordings on the patient’s medical record may help guard against tampering or misrepresentation in the case of a malpractice lawsuit	An original copy of all audio-recordings made on the app should be stored in the appropriate patient’s electronic medical record, or in a secure location that is accessible by medical record staff
4. Clear legal responsibilities	Patients using the app must be aware that they are legally responsible for the safety of the audio-recordings that are saved on and shared from their mobile, just as they are responsible for any copy that they are given of any component of their medical record	Include statement of responsibility on the opening screen of the app and in all app promotion material
5. Minimal upkeep	Once developed and implemented into usual care, the app should require minimal input from the staff and minimal ongoing financial costs	Integrate the app into existing hospital procedures; Automate processes where possible (eg, automatic upload of recordings from the app to the medical record); Use the latest secure cloud infrastructure to keep ongoing costs down
6. Minimal upfront costs	Additional funding could not be sought until the app had been piloted in a clinical setting and evidence was obtained about the usability of the app, whether it met requirements 1 to 5, and the extent of uptake among patients	Develop a minimal viable product. Results of the pilot can then be used to refine the product and support further, ongoing funding; Develop in iOS only (not Android); Delay investing in automating processes until after piloting

**Table 2:** The number and type of attendees at each workshop.

Category	Workshop 1	Workshop 2	Workshop 3	Workshop 4	Workshop 5	Workshop 6
Researcher	2	2	2	2	2	2
App developer	2	2	2	2	2	2
Consumer	2	1	1	0	0	3
Information technology	1	0	0	0	0	1
Oncologist	0	0	0	0	0	1
Nurse	0	0	0	0	0	1
Allied health	0	0	0	0	0	1
Medical records	0	0	0	0	0	1
Hospital volunteer	0	0	0	0	0	1
Total	7	5	5	4	4	13

### Outcomes From Workshops 1 to 4

In answer to question 1 (“What do we want the app to do?”), the attendees took a blue sky thinking approach and articulated an ultimate aim for the app, as well as a list of all possible functions. The ultimate aim of the app was unanimously decided and described as “Improve the quality of patients’ care,” that is, improving the quality of patients’ participation, understanding and support during treatment, diagnosis, decision making, and support during their cancer journey. This was used as a keystone upon which to design the app and guide decision making regarding design, functionality, and utility. The possible functions identified by the attendees included the following: audio-record, share audio-recordings, listen back to audio-recordings, use without help, secure, categorize and label audio-recordings, send audio-recordings to Peter Mac, make notes, and read notes.

Table 3 summarizes the potential pitfalls and corresponding preventative strategies that were identified by the workshop attendees in answer to question 2 (“How can we imagine the app failing?”). This exercise indicated that, to be successful, the app would need to be paired with a promotion and education strategy to teach patients how and when they should use the app, their rights and responsibilities regarding use and sharing of audio-files, and to build trust with clinical staff.

**Figure 2:**
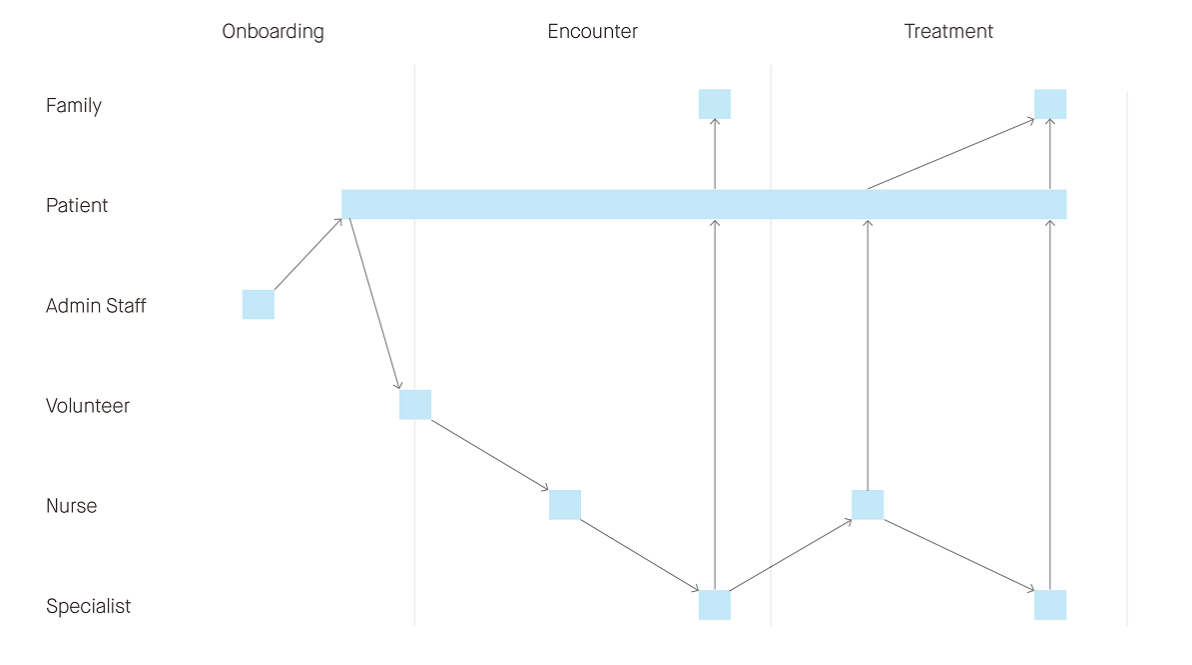
A journey map representing the envisaged pattern of use of the SecondEars consultation audio-recording app.

In answer to question 3 (“Who will be involved in using the app?”), the attendees mapped out a typical patient journey within the hospital, identifying the key interactions that all personnel would have with each other and the app (see Figure 2).

**Table 3:** The potential pitfalls, knowledge-seeking questions, and preventative strategies outlined in workshops 1 to 3.

Potential pitfalls	Knowledge-seeking question	Preventative strategy
1. The app is too difficult to use	How do we make the app intuitive to the patient, the carer, and the health care community?	User-friendly, simple design; Education on how to use (provided with appointment booking information); Volunteer assistance in clinic
2. The app leads to incidents of personal damage (eg, security breaches)	How do we gain and maintain trust?	Appropriate security infrastructure; Education on responsible sharing (presented at app log-in); Upload to medical record required before play back or sharing
3. Patients do not download the app	How do we support appropriate and wide distribution?	Promotion (notification with appointment booking, signs in waiting room, and encouragement from the staff)
4. Patients forget to use the app	How do we let everyone know when it is the right time to use the app?	Promotion (notification with appointment booking, signs in waiting room, and encouragement from the clinical staff)
5. Patients do not find the app useful	How do we align the service to the benefits of audio-recordings that have already been established through research?	Draw on existing research; Include consumers in development

**The features that could be included in the SecondEars app and their corresponding prioritization according to the MoSCoW (Must have, Should have, Could have, and Won’t have) method.**

Must have

- Record consultation
- Upload required before playback
- Playback recording
- Patient identification number required for log-in
- Explain legal context and provide instructions
- Delete recording
- Share recording
- Recording library (list)
- Peter Mac access to data (list of recordings to download and attach to medical record)

Should have

- Notes on playback (editable)
- Notes while recording (view only)
- Categorize recordings (colors or tags)
- Ability to associate each recording with the relevant clinician (ie, tag recording as Physio, doctor, and nurse)

Could have

- Capture next appointment
- Reminder notification of next appointment

Won’t have

- Authenticate and user management
- Booking system integration
- Push notifications and reminder emails
- Upload over 3G, Wi-Fi setting (user controlled)
- Barcode or quick Response code scan
- Access recording and share recording without device

Figure 2 shows that the initial promotion of the app would need to be undertaken by the administration staff. Information about the app would be provided to the patients by the administration staff when their first appointment is booked. The patients would then download the app before their first consultation (or “encounter”) at the hospital. Hospital volunteers, who are a regular presence at Australian hospitals, would be made aware of the app and be available to assist patients in downloading it while the patients wait for their appointment. The patients may then use the app to audio-record the nurse and/or specialist during their first consultation and at subsequent consultations. The pattern of use also shows that the patients may share the audio-recordings with their family members or friends after their hospital encounter. According to the Theory of Planned Behavior, integrating the app into the clinical process could influence subjective norms and therefore influence the patients’ behavior.

During workshops 1 to 3, additional features were suggested as complements to the audio-recording functionality in the app, and suggestions were made regarding how patients could label, categorize, store, share, and search for audio-recordings once they are made. These features are listed in Textbox 1 along with their priority according to the MoSCoW exercise that was completed in Workshop 4. The features classified as “Must have” or “Should have” were considered within the scope of the app. These features were included in the wireframe of the app, which was trialed in workshops 5 and 6.

### Outcomes of Workshops 5 and 6

Feedback from workshop 5 informed the development of the wireframe, which was presented on an iPhone in workshop 6. The members of the co-design team attended workshop 6, and decisions from workshops 4 and 5 were reviewed at the start of the workshop. A total of 9 members of the co-design team trialed the wireframe of the app in workshop 6 (see Table 2). The 2 researchers and the 2 app developers did not trial the wireframe in this workshop as they had viewed the designs in workshop 5 and were not the intended users of the app. The wireframe contained all app content but without the colors, etc, of a final version. All attendees completed the 4 tasks without prompting. Their feedback suggested that the app was “quite straightforward” to use (consumer). Suggestions were also made to further improve usability. A clinician suggested that the font size be increased (“I couldn’t read that without my glasses on”); therefore, the app was adjusted to automatically match the font size settings on the user’s phone. Another clinician suggested placing the *play* symbol (a triangle) inside a circle so that it looked more like a button. Moreover, 1 clinician and 1 consumer suggested that app users should be able to navigate back to the instructions page if they want a reminder of how to use the app (the wireframe displayed the app-use instructions only once, immediately after log-in). The representative from medical records suggested an amendment to the terms and conditions. Several attendees suggested adding an open category in the labeling function so that patients can assign their own labels to recordings or note if more than 1 clinician was present at the appointment.

Some extra features were also suggested, which were shelved for later iterations. A clinician suggested allowing patients to attach photos to audio-recordings; she often draws pictures to explain medical procedures to patients or shows them scans that they may want to photograph. Several people suggested changing the notes feature so that the user could create notes that were not related to a specific audio-recording or, conversely, to link notes to a particular section (ie, minute and second) of an audio-recording.

After prioritizing all the feedback generated from workshops 5 and 6, the wireframes were adjusted to enhance the navigation and layout. The hierarchy of the individual recordings screen was reconsidered to place a stronger focus on the core feature of listening to an audio-recording. Additional smaller changes were made to increase the overall accessibility of the app, resulting in an experience that was more intuitive and easier to navigate.

### User Acceptance Testing

Wave Digital incorporated the feedback from workshops 5 and 6 into the visual interface design of the app to develop a prototype for user acceptance testing. Unlike the wireframe, this prototype contained all the design features and was, in essence, a complete app. Overall, 7 people tested this prototype: 2 clinicians, 4 researchers, and 1 consumer. Feedback included the following: bugs or defects (eg, typos or unexpected error messages), design-related feedback (eg, recommendations for consistency of the *exit* and *back* buttons), suggestions for changes to written content or copy, and feedback relating to the user interface for the Amazon Web Server (eg, allowing audio files to be deleted by the administrator).

### The Final App Design

The feedback from user acceptance testing was incorporated into a final version of the app. The design of this version of the app is included in Multimedia Appendix 1 and its functionality is listed in the *Must have* and *Should have* sections of Textbox 1.

## Discussion

### The SecondEars App

The SecondEars consultation audio-recording app for cancer patients was successfully co-designed and a prototype was developed in iOS. This mHealth patient-identified solution has been designed to facilitate its implementation in a clinical setting and has been developed within a framework of the Theory of Planned Behavior. The app enables a copy of each audio-recording to be saved on the appropriate patient’s medical record, thereby allowing the hospital to retain access to the original recording for medico-legal reasons. This balance between patient autonomy and clinician security was achieved through stakeholder engagement, co-design workshops, and user acceptance testing to ensure that SecondEars was designed to meet the requirements of all users. Furthermore, the app was designed to have low upkeep and minimal burden on clinical processes.

### Principal Findings

Most of the requirements identified through stakeholder engagement echo the findings from Moloczij et al [15] and van Bruinessen et al [11] regarding barriers and facilitators to implementation (summarized in the Introduction of this paper). Stakeholders in this study emphasized the importance of minimizing upfront costs. This requirement led to the development of a pragmatic, minimum viable product comprising only the essential core features. The first co-design workshop confirmed that the aim of the app is to “improve patient care.” This impetus, and the MoSCoW session in workshop 4, worked to focus the app development on the most important features: audio-recording and sharing the audio-recording securely and confidentially. Paring back the app to contain only essential features ensures will help to minimize upkeep and keep the app cost-viable for a public health care setting. Further features can be adapted and expanded in the future as the feasibility and efficacy of the app become established through the evaluation of implementation in a clinical setting.

SecondEars was designed to strike a balance between 2 imperatives that, at times, could come into conflict: patient autonomy over the audio-recording and legal protection for the clinician. Our stakeholders identified that medical information provided by the doctor would form a part of the medical record, which prompted a solution that met both of these imperatives: compulsory uploading of audio-recording before playback and sharing would provide the clinician with a measure of security while maintaining the patient’s control over the creation and distribution of the audio-recording.

Furthermore, useful data were generated regarding practical recommendations to facilitate implementation after piloting. The second and third co-design workshops confirmed that the app would need to be distributed with publicity and education information to ensure timely uptake of the app, which is in line with the Theory of Planned Behavior. The patient journey map revealed how many different types of people would encounter the app, suggesting that an app can be a means to change subjective norms.

A recent systematic review found that health care apps are more likely to be effective if they are user-friendly and require minimal time investment [26]. This is in line with the perceived behavioral control aspect of the Theory of Planned Behavior; people will be more likely to take up a behavior if they feel that they do not have external time pressures and if they believe that they are capable of the behavior. User acceptance testing demonstrated that testers found the app easy to use. The app is currently being piloted in a wider patient population in a clinical setting to determine feasibility and ease of use.

### Limitations

This study’s strength lies in its theoretical basis and the extent of stakeholder and consumer engagement. The researchers chose to include multiple stakeholders and not just consumers. This had benefits in terms of practical recommendations and requirements for the app. However, the inclusion of multiple stakeholder groups meant that the number of people in each group had to be limited to maintain a manageable number of attendees for the workshops. The contributions of consumers were emphasized throughout (there was a higher proportion of consumers on the co-design team compared with the other stakeholder groups); unfortunately, because of scheduling conflicts, the MoSCoW session in workshop 4 was attended by only the researchers and app developers. Future co-design research should ensure that the entire co-design team is involved when features are prioritized. Future studies could also choose to repeat workshops to provide more opportunities for consumers to be involved. This approach would increase the number and diversity of the consumers in the co-design team (eg, wider range of ages, ethnicities, and time since diagnosis), but it may result in each consumer individually having less input overall.

Financial and pragmatic constraints identified through stakeholder engagement mean that the app was developed for iOS only, not Android. This may introduce a perceived external control for patients who do not have the necessary equipment to use the app, which, according to the Theory of Planned Behavior, may negatively affect behavior change. The number of patients who are ineligible to participate in piloting because of not having an iPhone will be recorded. The drawbacks of having the app on only 1 platform will be temporary as the app will be adapted for Android once the current design has been piloted in a clinical setting.

Although the Theory of Planned Behavior is generally a well-accepted theory of behavior change and has been used to design many behavior change interventions, there have been inconsistent results regarding its effectiveness, [27,28] and debate continues around the use of the theory in this context [29,30]. Future researchers can refer to a recent report to consider the full suite of options for behavior change frameworks for intervention development [31].

### Comparison With Previous Work

To the authors’ knowledge, no other consultation audio-recording apps have been developed via a co-design approach. Previous research has successfully used the Theory of Planned Behavior as a guide for developing health care interventions [32], and other apps have successfully been developed following a behavior change theory [33].

Some of the desired features, such as the app’s automated integration with the medical record, are very complex. Other studies have also encountered similar problems when trying to integrate an app with the medical record [34]. The pragmatic choices made in this project will allow the app to be piloted in a clinical setting before significant investment is made to integrate it with the medical record.

### Future Research

The SecondEars app is currently being piloted with patients in a clinical setting. Feedback from patients and clinicians will inform any further design changes that need to be made before implementing the app as part of usual care at Peter Mac. Piloting will also inform the education and promotion strategy that was identified as important during the development process. Upon implementing the app, data could be collected regarding behavior change (uptake, use, etc) to evaluate the success of the app.

Clinical staff have expressed concern regarding potential changes in communication caused by the act of recording a consultation, such as loss of rapport-building, and reduced personalization of information delivery, as fear of litigation may drive information delivery rather than patient need [6]. Longitudinal evaluation of SecondEars implementation could identify whether these changes occur and whether they are sustained as the app becomes a familiar and routine component of care. Longitudinal evaluation could also provide opportunities to study the impact that the SecondEars app has on patients’ recall and understanding of medical information and their participation in clinical decision making.

Future versions of the app should include interface options for specific patient groups, such as translations and adaptations for culturally and linguistically diverse patients, and text-to-voice options for patients who are visually impaired. Furthermore, there are potential, unexplored benefits to the SecondEars app that could be investigated in future studies. For example, the consultation audio-recordings could provide professional development opportunities for clinicians or teaching opportunities for clinical students. Other studies have also discussed the potential cross-professional use of consultation audio-recordings as a helpful mechanism of information transmission between primary and tertiary settings [6].

### Conclusions

The SecondEars app has been designed to be a viable and cost-effective means of integrating consultation audio-recordings into an oncology setting. The app embraces existing technology as a patient-driven solution to improve patient-centered care. Engagement of stakeholders and consumers in the co-design process ensured that barriers to implementation were addressed and facilitators were leveraged. The SecondEars prototype is currently being piloted with patients in a clinical setting before implementation.

## Appendix

Multimedia Appendix 1 Screenshots of the SecondEars app design.

## Abbreviations

IT: information technology
mHealth: mobile health
