# Supplementary material for: Co-Design of a Consultation Audio-Recording Mobile App for People With Cancer: The SecondEars App
Source: JMIR Form Res. 2019 Mar 12;3(1):e11111. doi: 10.2196/11111 (PMC6434400; doi:10.2196/11111)
Supplement: Multimedia Appendix 1 [file formative_v3i1e11111_app1.pdf]

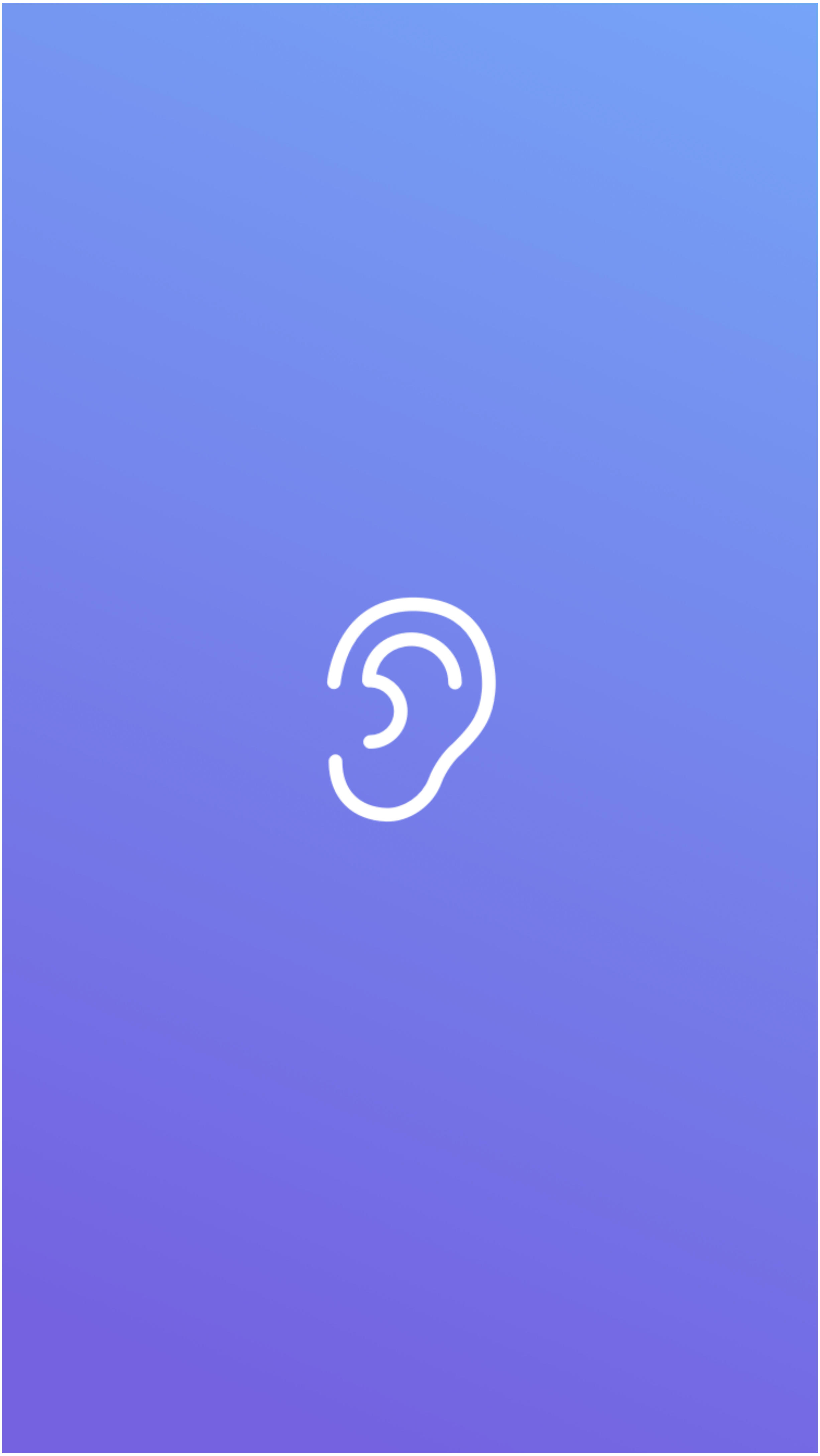

●●●●● Carrier 9:41 AM 100%

Second Ears

The Peter MacCallum Cancer Centre's Second Ears app helps you record and recall conversations between you and your clinicians. You never have to worry about missing something important, and you can share it with your family and carers too.

Start

Powered by wave

●●●●● Carrier 9:41 AM 100%

Peter Mac
Peter MacCallum Cancer Centre
Victoria Australia

PURN ?

Full Name

Date of Birth

Sign in

Help

Powered by wave

●●●○○ Carrier 9:41 AM 42%

Instructions Done

1. Record

When you go in to your consultation use the Second Ears app to record your conversation.

2. Upload

After you've finished recording it will need to be uploaded to the hospital. Choose when it suits you to upload.

3. Listen

Once the recording is uploaded you can listen to your recording and take notes to help you remember information.

4. Share

Play the recording to family members and carers, or send them a copy too.

Remember: This recording is your responsiblity once shared outside of the Second Ears app - much like if you took a copy of your medical record outside of the hospital!

Powered by wave

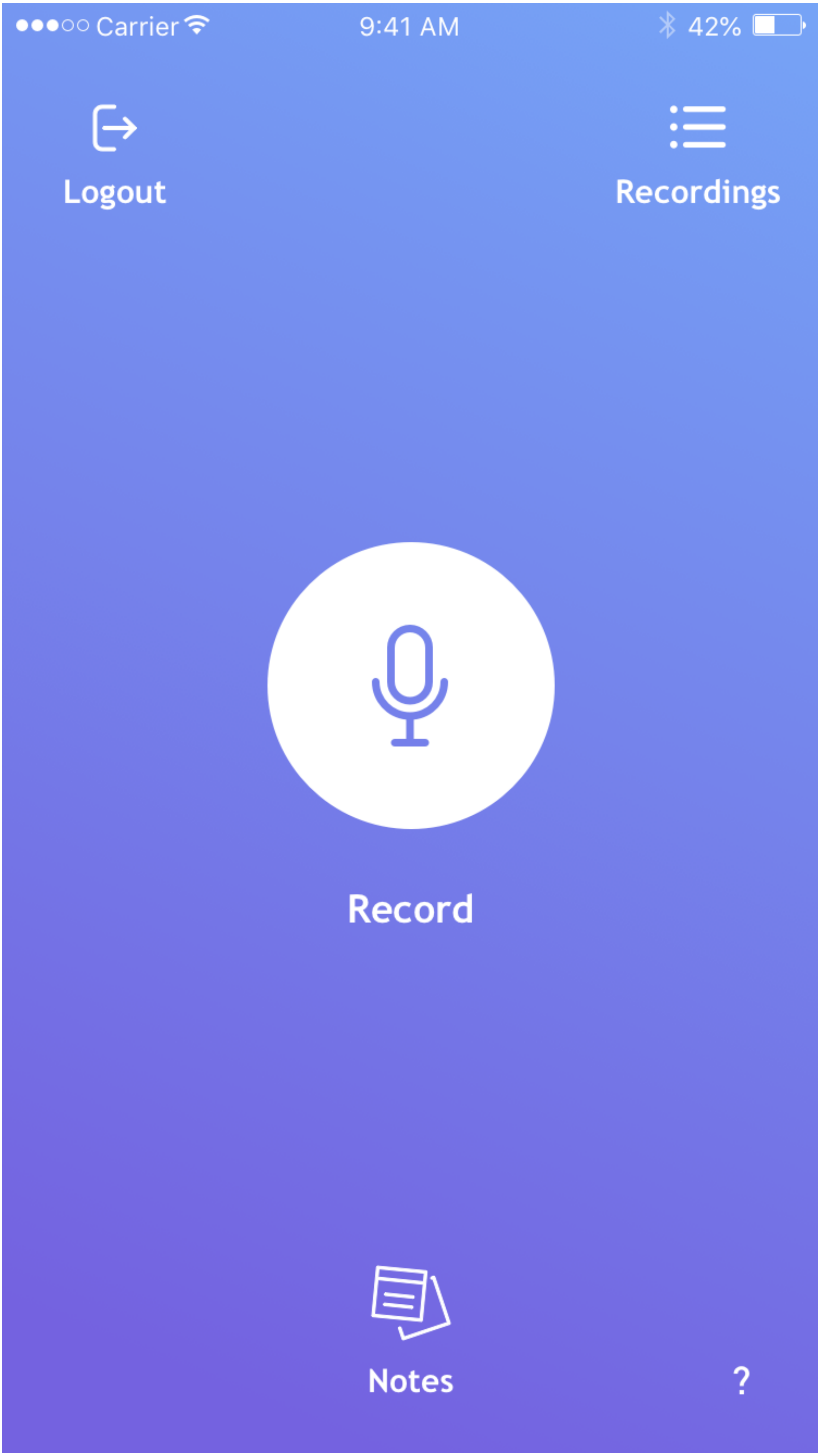
●●●○○ Carrier
9:41 AM
42%
Logout
Recordings
Record
Notes
?

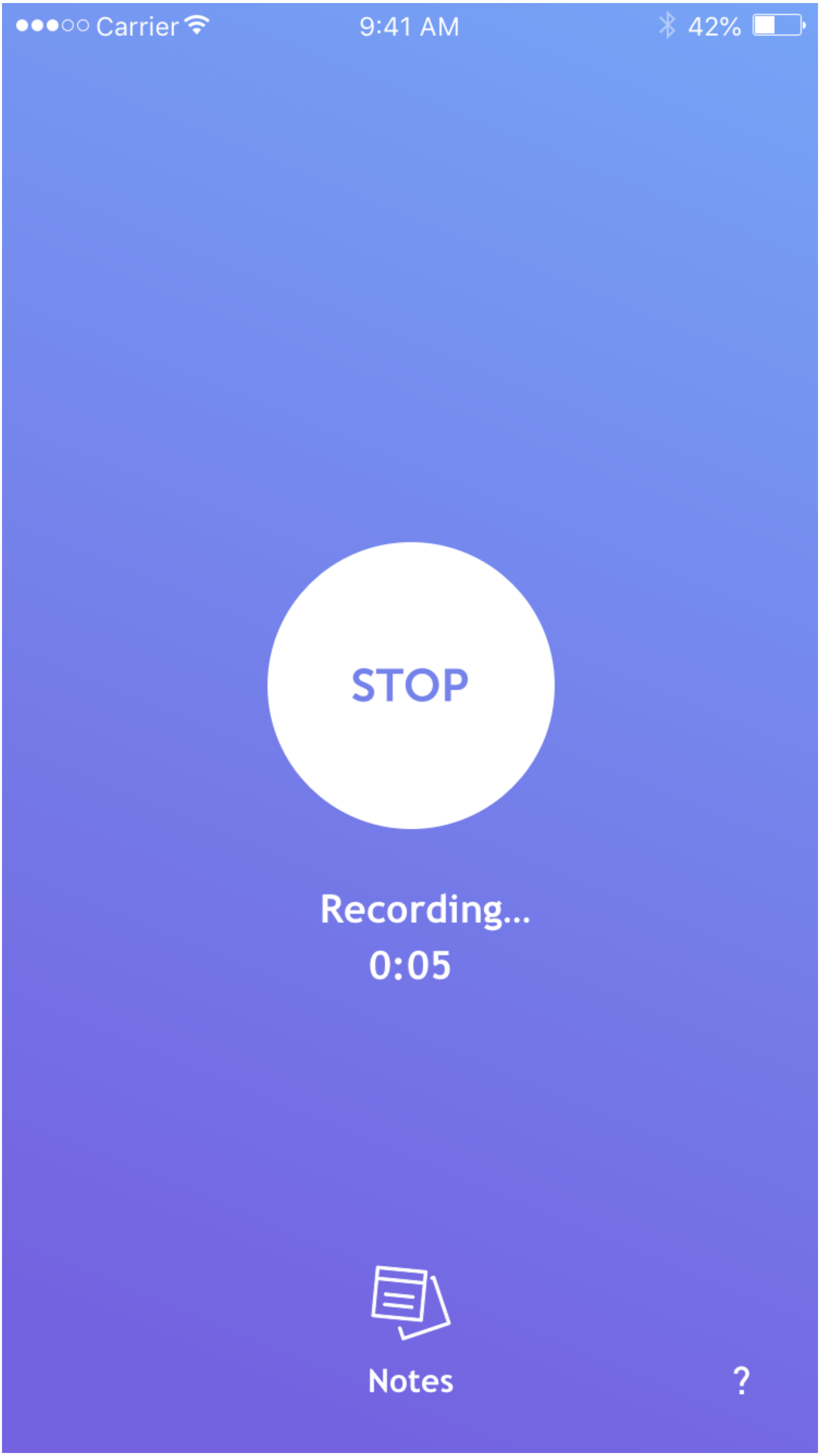
●●●○○ Carrier
9:41 AM
42%
STOP
Recording...
0:05
Notes
?

●●●○○ Carrier 9:41 AM 42%

Notes

23 May 2017 – 10:08 AM

Dietician

Lorem ipsum dolor sit amet, consectetur adipiscing elit. Vestibulum nisl nulla, facilisis sed massa eu, vestibulum posuere.

---

23 May 2017 – 9:26 AM

Radiologist

Lorem ipsum dolor sit amet, consectetur adipiscing elit. Vestibulum nisl nulla, facilisis sed massa eu, vestibulum posuere mi. Morbi eu augue rhoncus, lobortis erat nec, tempor magna. Nam molestie rhoncus.

---

18 March 2017 – 12:32 PM

Physio

×

Close

●●●○○ Carrier 9:41 AM 42%

Recording 0:05

23 May 2017 – 10:08 AM

Dietician

Lorem ipsum dolor sit amet, consectetur adipiscing elit. Vestibulum nisl nulla, facilisis sed massa eu, vestibulum posuere.

---

23 May 2017 – 9:26 AM

Radiologist

Lorem ipsum dolor sit amet, consectetur adipiscing elit. Vestibulum nisl nulla, facilisis sed massa eu, vestibulum posuere mi. Morbi eu augue rhoncus, lobortis erat nec, tempor magna. Nam molestie rhoncus.

---

18 March 2017 – 12:32 PM

Physio

Close

Back **Recordings**

23 May 2017 - 10:08 AM
Dietician

23 May 2017 - 9:26 AM
Radiologist

18 March 2017 - 12:32 PM
Physio

2 January 2017 - 10:02 AM
Nurse

12 December 2016 - 3:23 PM
Dietician

●●●●● Carrier 9:41 AM 100%

Back **Recordings**

1 June 2017 - 9:28 AM UPLOAD

23 May 2017 - 10:08 AM
Dietician

23 May 2017 - 9:26 AM
Radiologist

18 March 2017 - 12:32 PM
Physio

2 January 2017 - 10:02 AM
Nurse

12 December 2016 - 3:23 PM
Dietician

Carrier 9:41 AM 100%

Back **Recordings**

1 June 2017 - 9:28 AM

23 May 2017 - 10:08 AM
Dietician

23 May 2017 - 9:26 AM
Radiologist

18 March 2017 - 12:32 PM
Physio

2 January 2017 - 10:02 AM
Nurse

12 December 2016 - 3:23 PM
Dietician

●●●●● Carrier 9:41 AM 100%

Back **Recordings**

1 June 2017 - 9:28 AM

23 May 2017 - 10:08 AM
Dietician

2017 - 9:26 AM
ist

Delete

18 March 2017 - 12:32 PM
Physio

2 January 2017 - 10:02 AM
Nurse

12 December 2016 - 3:23 PM
Dietician

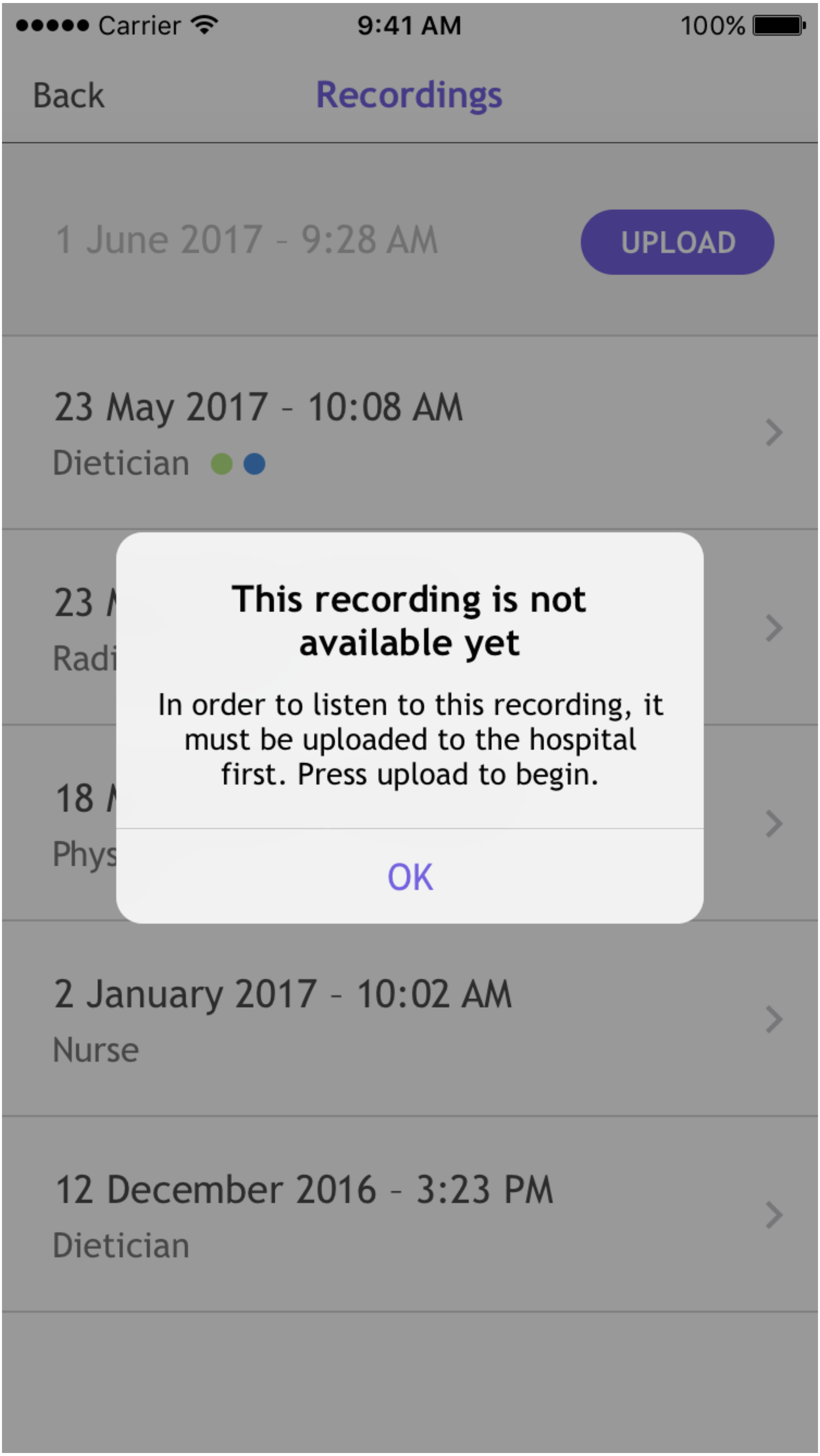

Carrier
9:41 AM
100%
Back
Recordings
1 June 2017 - 9:28 AM
UPLOAD
23 May 2017 - 10:08 AM
Dietician
This recording is not available yet
In order to listen to this recording, it must be uploaded to the hospital first. Press upload to begin.
OK
2 January 2017 - 10:02 AM
Nurse
12 December 2016 - 3:23 PM
Dietician

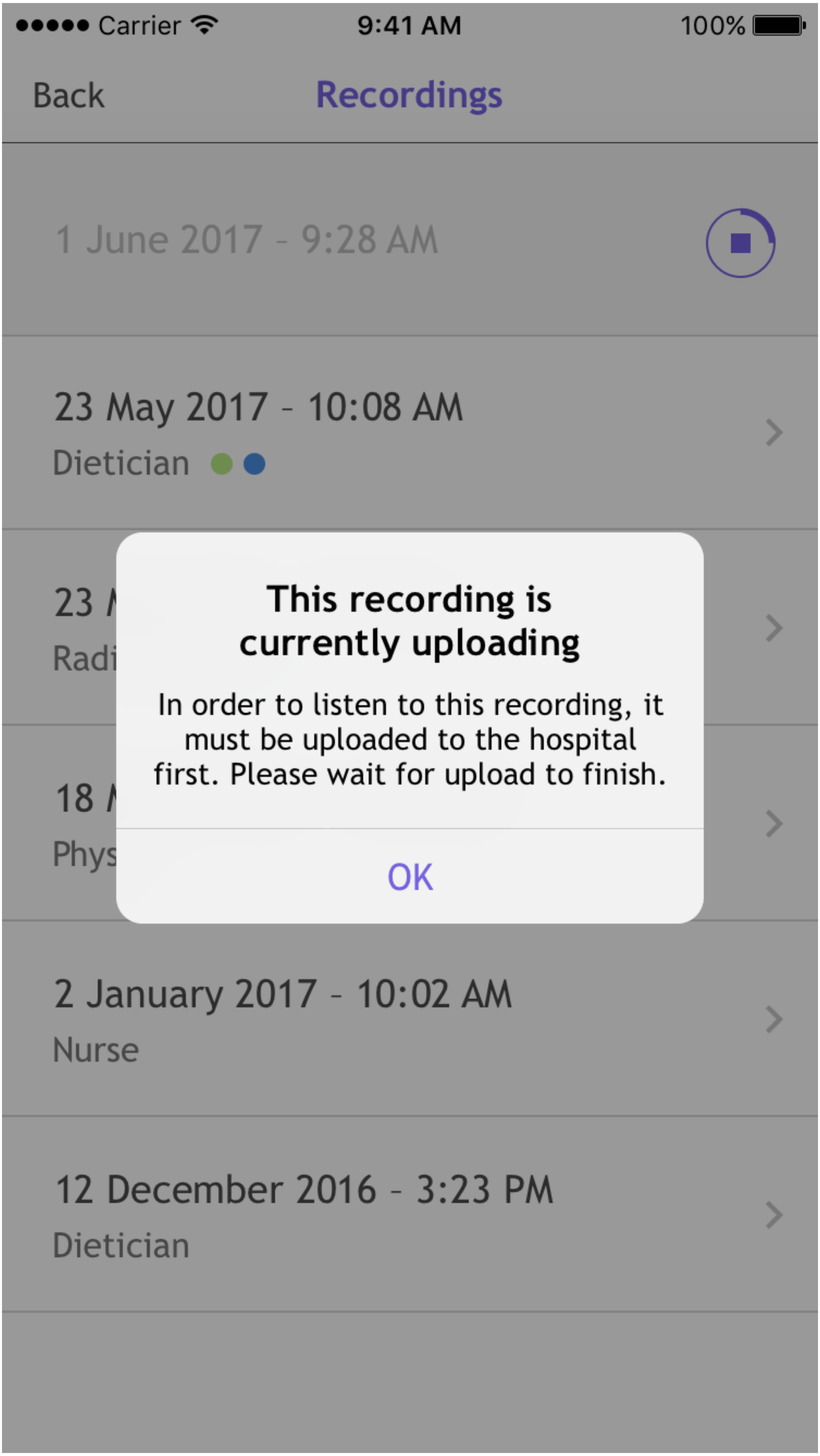

●●●●● Carrier
9:41 AM
100%
Back
Recordings
1 June 2017 - 9:28 AM
23 May 2017 - 10:08 AM
Dietician
23 M
Radi
18 M
Phys
This recording is currently uploading
In order to listen to this recording, it must be uploaded to the hospital first. Please wait for upload to finish.
OK
2 January 2017 - 10:02 AM
Nurse
12 December 2016 - 3:23 PM
Dietician

Back **Recordings**

1 June 2017 - 10:08 AM

16:12 -19:48

Share

Delete

Who Dietician >

Tags >

Notes

Enter your notes here...

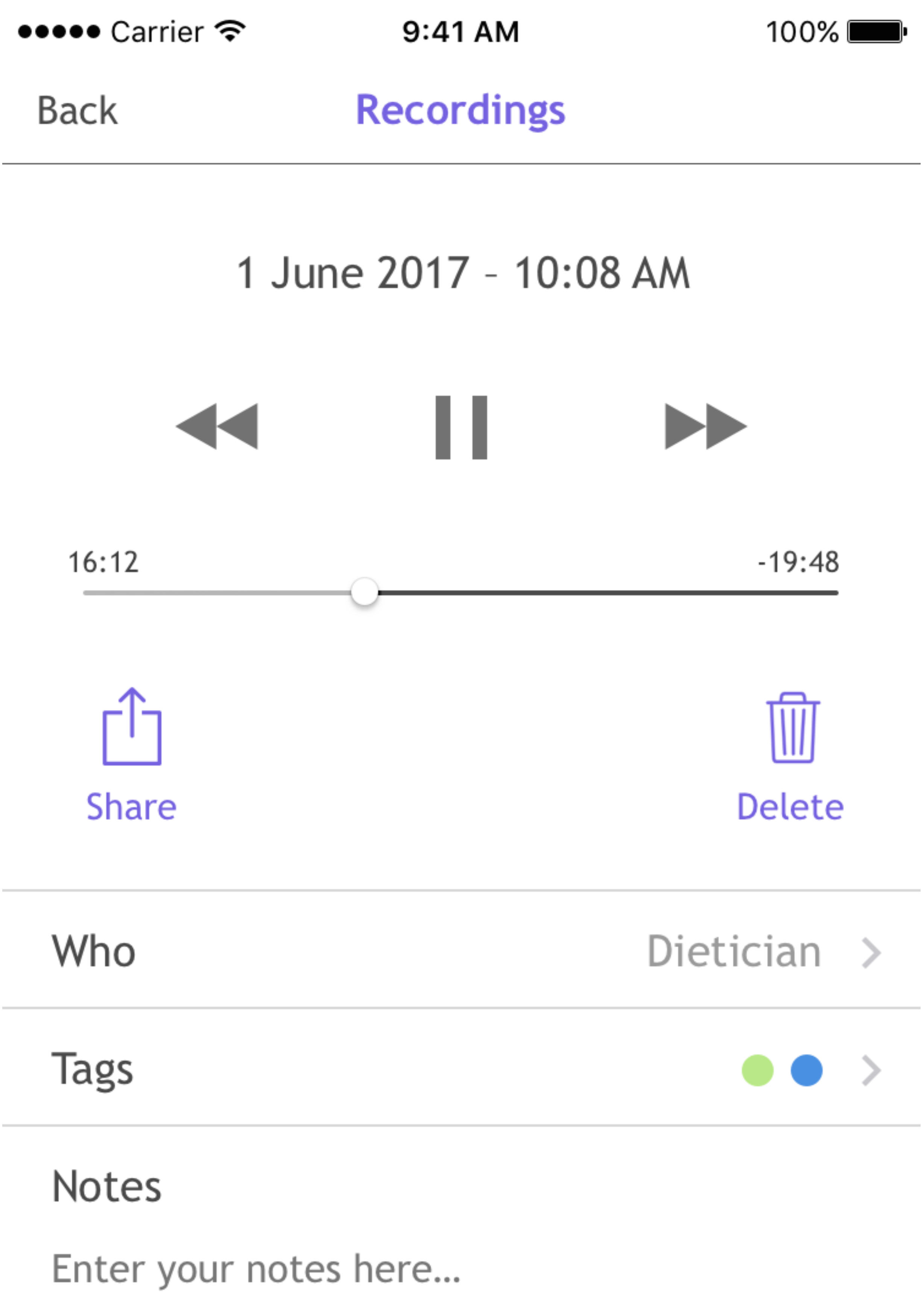

Carrier
9:41 AM
100%
Back
Recordings
1 June 2017 - 10:08 AM
16:12
-19:48
Share
Delete
Who
Dietician
Tags
Notes
Enter your notes here...

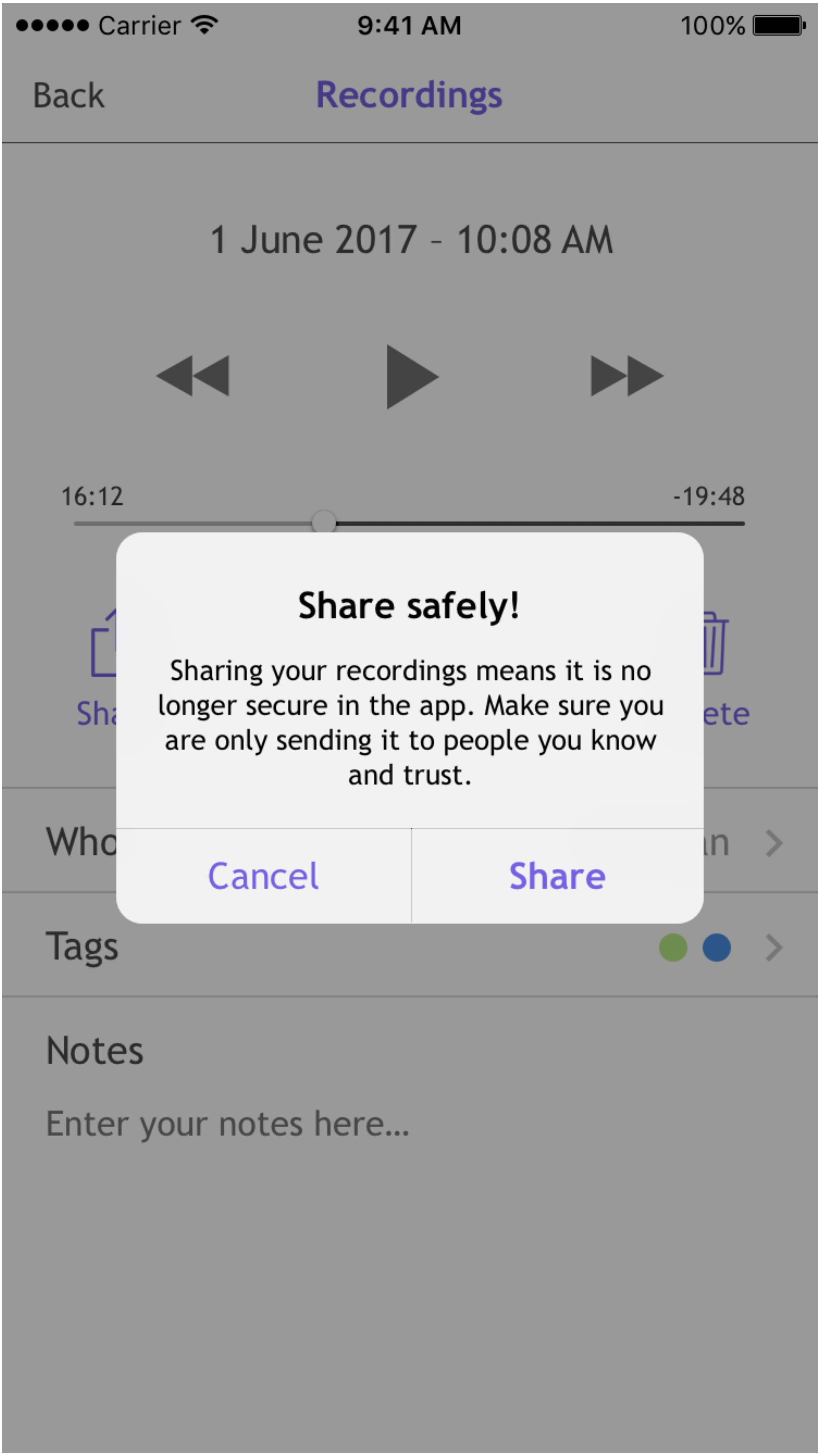
Carrier
9:41 AM
100%
Back
Recordings
1 June 2017 - 10:08 AM
16:12
-19:48
Share safely!
Sharing your recordings means it is no longer secure in the app. Make sure you are only sending it to people you know and trust.
Cancel
Share
Tags
Notes
Enter your notes here...

●●●●● Carrier 9:41 AM 100%

Back **Who**

Doctor

Nurse

Pharmacist

Social Worker

Physio-therapist

Psychologist

Dietician

Radiation Therapist

Other

●●●●● Carrier 9:41 AM 100%

Back

Who

Doctor

Nurse

Pharmacist

Social Worker

Physio-therapist

Psychologist

Dietician

Radiation Therapist

Other

●●●●● Carrier 9:41 AM 100%

Back **Tags**

ADD TAG

- Red
- Orange
- Yellow
- ✓ Green
- ✓ Blue
- Purple
- Grey
